# Is the Peer Presence Effect on Heightened Adolescent Risky Decision-Making only Present in Males?

**DOI:** 10.1007/s10964-019-01179-9

**Published:** 2019-12-20

**Authors:** Ivy N. Defoe, Judith Semon Dubas, Edwin S. Dalmaijer, Marcel A. G. van Aken

**Affiliations:** 1 University of Amsterdam, Postbus 15776, 1001 NG Amsterdam, The Netherlands; 2 Developmental Psychology, Utrecht University, Utrecht, The Netherlands; 3 MRC Cognition and Brain Sciences Unit, University of Cambridge, Cambridge, United Kingdom

**Keywords:** Risky decision making, Risk behavior, Adolescence, Peer influences, Gender differences, Adolescent phase differences

## Abstract

Social neurodevelopmental imbalance models posit that peer presence causes heightened adolescent risk-taking particularly during early adolescence. Evolutionary theory suggests that these effects would be most pronounced in males. However, the small but growing number of experimental studies on peer presence effects in adolescent risky decision-making showed mixed findings, and the vast majority of such studies did not test for the above-described gender and adolescent phase moderation effects. Moreover, most of those studies did not assess the criterion validity of the employed risky decision-making tasks. The current study was designed to investigate the abovementioned hypotheses among a sample of 327 ethnically-diverse Dutch early and mid-adolescents (49.80% female; *M*_age_ = 13.61). No main effect of peer presence on the employed risky-decision making task (i.e., the stoplight game) was found. However, the results showed a gender by peer presence moderation effect. Namely, whereas boys and girls engaged in equal levels of risks when they completed the stoplight game alone, boys engaged in more risk-taking than girls when they completed this task together with two same-sex peers. In contrast, adolescent phase did not moderate peer presence effects on risk-taking. Finally, the results showed that performance on the stoplight game predicted self-reported real-world risky traffic behavior, alcohol use and delinquency. Taken together, using a validated task, the present findings demonstrate that individual differences (i.e., gender) can determine whether the social environment (i.e., peer presence) affect risk-taking in early- and mid-adolescents. The finding that performance on a laboratory risky decision-making task can perhaps help identify adolescents that are vulnerable to diverse types of heightened risk behaviors is an important finding for science as well as prevention and intervention efforts.

## Introduction

Most risk behaviors that peak in adolescence occur when adolescents are with their peers (Steinberg 2008). However, most studies on peer influences on adolescent risk-taking do not investigate the direct effect of presence of peers on heightened adolescent risk-taking (see: Defoe et al. 2015). Instead, past studies have often focused on similarity in risk-taking behaviors among peers, with the assumed mechanism being *social learning* (e.g., Haynie and Osgood 2005). Alternatively, current advances in adolescent brain research suggest that the mere “peer presence” (with or without social learning) leads to heightened adolescent risk-taking (Albert and Steinberg 2011). A small but growing number of experimental studies have investigated this hypothesized peer presence effect on adolescent risky decision making (e.g., de Boer et al. 2017; Gardner and Steinberg 2005; Somerville et al. 2019). However, the results have been inconsistent thus far (for a review see: Defoe et al. 2019), the vast majority of those studies did not consider gender and/or adolescent phase moderation effects and the criterion validity of the employed risky decision-making tasks has rarely been assessed. Hence, the current study adds to the literature by investigating the above-described peer presence effects on risky decision making using a large sample of early-middle adolescents (*N* = 327), which further enabled the testing of gender and/or adolescent phase moderation of such peer presence effects (study 1). In doing so, the criterion validity of the employed risky decision-making task (“the stoplight game”; Chein et al. 2011) was also investigated by examining whether performance on that task is associated with real-world risk taking (study 2).

*Neurodevelopmental imbalance models* postulate that heightened adolescent risk-taking occurs, particularly in emotionally arousing contexts, wherein adolescent’s hyper-responsive motivational-reward system in the brain gets triggered, resulting in a pronounced imbalance with their relatively immature cognitive control system (Somerville et al.^ 2011^; Steinberg 2008). Distinctively, social variants of neurodevelopmental imbalance models (i.e., *social neurodevelopmental imbalance models*) postulate that peers increase risk-taking particularly in adolescence, because the mere presence of peers activates the same brain regions as rewards do, and in that sense, peers can be considered as socially rewarding (Steinberg 2008). Similar sentiments have also been echoed, such as the idea of *unstructured socializing* among adolescents and its link to deviant behavior (Hoeben et al. 2016; Osgood et al. 1996). However, whereas some studies clearly showed that the mere presence of peers increases risky decision making in adolescents (e.g., Gardner and Steinberg 2005), other studies reported that mere peer presence does not increase risk-taking in adolescents (e.g., Kretsch and Harden 2014) and college students (ages 19–24; e.g., Nawa et al. 2008). Yet other studies showed mixed/conditional effects (Somerville et al. 2019) (for a review see: Defoe et al. 2019). These inconsistent results could perhaps be attributed to gender and/or adolescent phase moderation effects in the influence of peer presence on risk-taking, but thus far such moderation effects have been largely neglected.

### Gender Effects

The social neurodevelopmental imbalance model (Steinberg 2008) does not make any explicit predictions about gender differences in peer presence effects on heightened adolescent risk-taking, perhaps because males and females presumably undergo similar neurological development. Some of the most dominant theories that suggest gender effects in risk-taking are evolutionary theories. One of such theories is the *sociobiological theory of male competitiveness*, which posits that risk-taking is a primarily male phenomenon, and especially so when males are in presence of other male counterparts (Wilson and Daly 1985). This is because, males’ fitness is assumed to stem from success in social interactions, wherein competition could arise. Specifically: “In a sociable species such as our own, in which there are long-term consequences of success and failure in competition, mediated by rank and reputation, we furthermore expect an evolved inclination toward the social display of one’s competitive risk-taking skills, and again this should be especially a masculine trait” (Wilson and Daly 1985; p. 66). More contemporary evolutionary perspectives also acknowledge that for males especially, engaging in risky behaviors during adolescence (such as violent or delinquent acts) may serve a signaling function that one is tough or strong, enhancing one’s reputation or status in the group (Ellis et al. 2012). As for empirical research, correlational and observational studies indeed show that many (antisocial) risk-taking behaviors are gender specific, with males taking more risks than females on average (Moffitt and Caspi 2001; for a meta-analysis, see: Byrnes et al. 1999). Relatedly, self-report studies also show that males are less resistant to (antisocial) peer influence (Steinberg and Monahan 2007; Steinberg and Silverberg 1986). However, gender differences have largely been neglected in research using laboratory or computer assessments of adolescent risk-taking, and the findings have been mixed (see: Defoe et al. 2015).

Moreover, only a few studies have investigated gender-moderated peer influences on risk-taking (cf. Weerman and Hoeve 2012; for a review see: McCoy et al.^ 2019^). The limited (correlational) studies that do, show mixed results pertaining to whether males or females are more susceptible to peer influence leading to risk-taking (e.g., Piquero et al. 2005; Weerman and Hoeve 2012). Additionally, one of the few experimental studies that investigated gender moderation of peer presence effects, showed that adolescent boys engaged in more risk-taking than adolescent girls when they completed a risky task with peers, but not when they completed the task alone (de Boer et al. 2017); while a more recent study using the same task but electronic peer confederates rather than actual peers did not (Harakeh and de Boer 2019). Moreover, although another experimental study (i.e., Gardner and Steinberg 2005) found that in the presence of peers, males gave more weight to the benefits of risk taking—no gender moderated peer presence effects were found on the employed risk-taking task. However, an absence of gender moderation in the peer presence effects in Gardner and Steinberg (2005) could be due to power issues as a result of the relatively small sample of adolescent participants. Thus, although gender has consistently been shown to moderate risk-taking in the real-world, the limited amount of studies on gender-moderated peer-presence effects on adolescent risk-taking in the laboratory show mixed findings.

### Adolescent Phase Effects

The social neurodevelopmental imbalance model suggests that during early adolescence, at the onset of puberty, an imbalance occurs between the hyper-responsive reward processing regions and slowly maturing cognitive control regions, which triggers risk-taking (Somerville et al. 2010; Steinberg 2007). Extrapolating from this theory, it is to be expected that early adolescents would engage in more risks than older adolescents (Crone and Dahl 2012; Somerville et al. 2010). Indeed, a recent meta-analysis on laboratory risky decision making showed that 11–13 year olds engaged in more risky decision making than mid-late adolescents (14–19 years) (Defoe et al. 2015). However, it should be noted that this meta-analysis combined mid-adolescents with late-adolescents, and thus it cannot be concluded for sure whether early adolescents took more risks than mid-adolescents *or* late adolescents separately. Moreover, it should be noted that unlike the meta-analytic findings that are based on laboratory risk-taking—in the real-world, both mid- and late- adolescents engage in more risk-taking than early adolescents (Agnew 2003; Farrington 1986). Thus, although an imbalance in the brain around early adolescence might make an individual more vulnerable to engage in risks, apparently ecological factors such as increases in risk exposure as adolescents age ultimately contribute to higher risk-taking levels among older adolescents versus younger adolescents in the real-world (Defoe et al. 2015, 2019). However, in the laboratory where risk exposure is equal for all ages, younger adolescents engage in more risks perhaps because of delayed cognitive ability (e.g., inhibitory control) (Defoe et al. 2019). All in all, accounting for adolescent phase differences in risk-taking whether in the laboratory or the real-world is essential.

As for adolescent-phase moderated peer influences, early adolescence is expected to be the period of heightened susceptibility for peer influence as a result of a hypothesized neurodevelopmental imbalance caused by pubertal changes around that period (Somerville et al. 2010; Steinberg 2007). Namely, particularly during early adolescence, peer presence is hypothesized to amplify the rewarding aspects of risks, which prevents adolescents from exercising inhibitory control (Steinberg 2008; Chein et al. 2011). Consistent with this account, one correlational study showed that whereas resistance to peer influence (i.e., peer pressure resistance) remained stable from 10–14 years, it increased from ages 14–18 (Steinberg and Monahan 2007). Additionally, peer approval and conformity have also been shown to decrease during mid-late adolescence (Berndt 1979). As for laboratory studies, a recent experimental study that investigated age as a continuous variable, reported that whereas peer presence (or more specifically “the monitoring of adolescent’s actions by a peer”) had a stronger risk-increasing effect for young adolescents on a “cold deliberative” version of a task, it had a stronger risk-increasing effect for older adolescents on a “hot affective” version of the task (Somerville et al. 2019).

Considered together, most theories and correlational studies suggest that from mid-adolescence onwards individuals are more likely to be equipped to resist peer influence. However, the only laboratory study (i.e., Somerville et al. 2019) that could be located that investigated age differences in peer presence effects on risk-taking showed mixed findings. Thus, it would be of added value for the current experimental study, with a sample of 12–13 year olds (1st year high-school pupils) and 14–15 year olds (3rd year high-school pupils), to investigate whether adolescent phase moderates the hypothesized link from peer presence to risky decision making.

### Criterion Validity

Another gap in experimental studies utilizing behavioral risky decision-making tasks, is that, surprisingly, the majority of those studies rarely include measures of real-world risk-taking behaviors to account for criterion validity (but see e.g., Kim-Spoon et al. 2016). Nevertheless, behavioral risky decision-making tasks have been generally shown to correlate with behaviors related to risk-taking, such as sensation-seeking (e.g., Steinberg 2008). Only one study appears to have investigated whether the stoplight game is related to real world risk-taking (Kim-Spoon et al. 2016). Results showed that performance on the stop-light game was related to a composite score of alcohol, marijuana and smoking in late adolescents (17–20 years; *N* = 24; 25% female) but not in adults (31–60 year olds; Kim-Spoon et al. 2016). Hence, the authors concluded that the stoplight game might be a promising tool for studying underlying behavioral and neurobiological mechanisms of particularly *adolescent* health risk behaviors. The current study investigates whether the stoplight game predicts self-reported real-world risk behaviors, while controlling for age, gender and sensation seeking.

## Current Study

The current article investigates whether peer presence, gender and adolescent phase predict adolescent risk-taking on a laboratory task, and if the hypothesized peer presence effect is moderated by gender and/or adolescent phase (study one). Specifically, study one investigated whether adolescents engage in more risks when they completed the stoplight game together with two same-sex peers (i.e., peer condition) versus when they completed the stoplight game on their own in an alone condition. Based on *evolutionary theory*, it is expected that the peer presence effect would be stronger for boys, and based on *social neurodevelopmental imbalance models*, it is expected that the peer presence effect would be stronger during early adolescence (versus middle adolescence).

The second study in the current article focusses on the criterion validity of the stoplight game (i.e., validation study). In doing so, the current study builds on, and extends, the only study that has assessed the criterion validity of the stoplight game (i.e., Kim-Spoon et al. 2016). First, unlike Kim-Spoon et al. (2016) that tested late adolescents, the present study focusses on younger adolescents (i.e., early- and mid-adolescents). Secondly, in addition to alcohol use, marijuana and smoking, the present study investigates whether experimental risk-taking is related to self-reported risky traffic behavior and delinquency, while controlling for possible confounding effects of age and gender. Additionally, sensation seeking is controlled for, since it has consistently been shown to be related to real-world and laboratory (experimental) risk-taking (Schonberg et al. 2011). Real-world risk behavior is assumed to be the result of risky decision-making (Petraitis et al.^1995^; Reyna and Rivers 2008); hence it is hypothesized that risky decision making on the stoplight game would predict various forms of real-world risk behaviors.

## Methods

### Study One

#### Participants

Participants were drawn from the first wave of a prospective 3-year longitudinal study in the Netherlands that began in 2012 (Defoe et al. 2016). For this multi-informant study, questionnaire data were annually (years 2012–2014) collected from adolescents at schools, and a subsample of their parents and siblings. During the first wave of the data-collections, 602 adolescents took part during school hours at their schools, and they were either in the first or third year of middle level secondary educational tracks (advanced vocational and technical tracks). In addition, adolescents also engaged in experimental sessions. In the first wave, the majority of the adolescents (93.2%) indicated that they were born in the Netherlands with 61.6% identifying as Dutch, and the rest identified with various other ethnicities. Most of their parents (68.4%) were either married or living together and 24.8% were either divorced or separated. Roughly half of the adolescents (44.90% fathers; 46.5% mothers) were unaware of their parents’ highest level of completed education, in part because parents (11.0% fathers; 11.8% mothers) were born abroad, in countries where the educational tracks were not comparable to the Dutch system. Of the reported education levels, 6.7% of mothers and 6.4% of fathers did not complete a high school education, 35.8% of mothers and 28% of fathers completed a lower or middle level vocational training and 3.8% mothers and 10.5% of the fathers completed a university degree. Extensive demographic information is published elsewhere (Defoe et al. 2016).

As mentioned, the current studies use data from the first wave. This data was collected in the Fall-Winter of 2012. For both studies, a subsample of 331 participants (49.80% female) who completed the stoplight game were used, and analyses were based on 327 valid cases. Adolescents were 12–16 years old (*M*_age_ = 13.61; *SD* = 1.19), and they were either in the 1st (*n* = 139; 47.50% female) or 3rd (*n* = 192; 51.60% female) year of middle level secondary educational tracks.

#### Procedure

Participants were recruited from high-schools in six different regions in the Netherlands. After approaching the schools via telephone calls and emails, eight schools agreed to participate, totaling approximately 810 potential participants. Parents received information letters about the research project as well as dissent letters that could be returned to the schools if parents did not want their children to participate (i.e., passive consent forms were used). Of the 810 potential student participants, a total of 9.75% did not participate at wave 1, because (1) they did not receive parental permission to participate, or (2) the students refused to participate on their own, or (3) due to other conflicts, for example the students were absent or ill on the day of the data-collection (Defoe et al. 2016). During the data-collections, participants received both written and verbal instructions by trained research assistants. The first part of a data-collection session consisted of a digital questionnaire and one cognitive task. A break followed and in the second part adolescents then completed the stoplight-game and two cognitive tasks.

Adolescents were randomly assigned to perform the stoplight game either simultaneously in an alone condition (i.e., they completed the task in the same classroom, behind their own computer) or a peer condition in a separate room. Research assistants ensured that participants in the alone condition (*n=* 252; 49.2% female) sat as far away from each other as possible, and that they did not communicate with each other during the stoplight game. Participants wore headphones during the alone condition in order to prevent the other participants in the room from hearing the sound effects of the stoplight game, as those participants were also busy with their own experimental tasks. In the Peer condition, randomly selected participants were placed in groups of three same-sex peers from their class (*n* = 120; 40 groups; 52.5% female). In this condition, participants played the stoplight game, one after the other, and were allowed to communicate with each other about the game.

Schools varied in how much time was allowed to be used for the data-collections (90–120 min). In some schools there was insufficient time for the stoplight game and in the group condition, hence complete data primarily existed for the first or second participant. Consequently, a total of 79 (51.9% female) of the 120 participants in the peer condition fully completed the stoplight game. The remaining participants in the peer condition did not (fully) complete the stoplight game because of the time constraints mentioned above, but also due to random causes, which included technical difficulties (e.g., computer crashed).

### Measures

#### Risky decision-making

Risky decision-making was measured with a two-dimensional version of the stoplight game, which was programmed in OpenSesame (Mathôt et al.^ 2012^). The game was constructed such that participants viewed the roads with a birds-eye view, with their car driving upwards on the screen. During each intersection approach, the traffic light jumped from green to yellow. Participants could decide to either brake by pressing the space bar, or to continue driving by not responding. If they chose not to stop, a crash could occur in which another car (not visible during the approach) would drive into the participant’s car. Risk-taking was operationalized as the proportion of yellow stoplights for which the participant did not brake, i.e., “percentage risky decisions” (Chein et al. 2011). The parameters spaces employed were identical to those reported by Chein et al. (2011), supporting information): temporal distance between intersections varied randomly between 10 and 16 s, the car’s braking duration was set to 0.5 s, the time between onset of the yellow light and the car reaching the intersection varied between 2 and 4.5 s in five evenly spaced steps (2000, 2625, 3250, 3875, 4500 ms), the delay of waiting at the traffic light set to 3 s, and the penalty for crashing was set to 6 s. In four practice runs, the probability of crashing was set to 0%, and to 50% in the successive 20 experimental trials, resulting in an overall crash probability of 40%. The task was designed to variably induce risky decision making in participants, and to prevent them from finding an optimal strategy, thus the degree of risky or cautious behavior is determined by individual differences rather than task characteristics (Chein et al. 2011). Finally, before participants began the stoplight game, they were informed that a prize would be given to the person in their school who finishes the game the fastest.

#### Strategy of analyses

Descriptive analyses were conducted in SPSS. Main analyses were conducted in Mplus 7.11 (Muthén and Muthén 1998–2012), wherein dependency within the group triads was accounted for. Specifically, the “Type = COMPLEX” feature was used to adjust for standard error biases as a result of the clustered data (participants clustered in groups of 3) (Korendijk et al. 2012). A Full Information Robust Maximum Likelihood Estimator was used for all models (Satorra and Bentler 1994) to adjust for possible deviation from normality and to allow the inclusion of incomplete data. Additionally, any item-missing data were dealt with using Full Information Maximum Likelihood (FIML) (Muthén and Muthén 1998–2012).

The analyses in Mplus were carried out using (ANOVA-analog) path models; that is, regressions with dummy-coded variables were used (Pehazur 1997). For the main effects model investigated whether peer presence, gender and adolescent phase showed main effects on risky decision making. That is, it was investigated whether adolescents took more risks in the peer condition (vs. alone condition), and whether there were gender and adolescent phase differences in risky decision making. Gender was coded as males = 0, and girls = 1. As for adolescent phase, as mentioned earlier, participants were between the ages of 12–16 years, and they were either in their first or third year of secondary school. Most participants in their 1st year of secondary school were between 12–13 years and most participants in their 3rd year of secondary school were between the ages 14–15 years. Thus consistent with these cohorts, 12–13 year olds (early adolescents) were coded as 0, and the remaining 14–16 year olds (mid-adolescents) were coded as 1 for the adolescent phase variable.

The first interaction model tested whether there was a two-way interaction effect for peer presence and gender, while controlling for adolescent phase effects. Similarly, in the second interaction model tested whether there was a two-way interaction between peer presence and adolescent phase, while controlling for gender effects. For the interaction terms the dummy variables were multiplied and added to the model simultaneously with the main effects. All models were just-identified with a perfect fit.

### Study Two

#### Participants and procedure

Please see study 1, as the same participants and procedure were used in study 2.

### Measures

#### Risky decision-making

A two-dimensional version of the stoplight game, which was programmed in OpenSesame (Mathôt et al. 2012) was used to assess risky decision making. Please see study 1 for details.

#### Risky traffic behavior

Three questions that were adapted from previous studies (Feenstra et al. 2002; van Nieuwenhuijzen et al. 2009) were used to assess risky traffic behavior. An example item is: “How often in the past four weeks, have you crossed a red light on your bike?”. Answer categories ranged from 0 = never to 4 = very often. A mean score was computed with higher scores indicating more risky traffic behavior. Cronbach’s alpha was 0.65 denoting adequate reliability.

#### Smoking

Smoking was measured with the question “Do you smoke tobacco? (cigarette, cigar, shag, (water-)pipe)?” that was derived from previous studies (e.g., Monshouwer 2008; Monshouwer et al. 2004; van Nieuwenhuijzen et al. 2009; Reijneveld et al. 2003). Answer categories ranged from 0 = No, I have never smoked to 5 = Yes, every day.

#### Alcohol use

Alcohol use was measured with a question that was adapted from previous studies (e.g., Monshouwer 2008, van Nieuwenhuijzen et al. 2009), namely “Do you drink alcohol?”. Answer categories ranged from 0 = No, I have never drunken alcohol to 5 = Yes, every day.

#### Marihuana use

Marihuana use was assessed with a question that was similar to marijuana questions used in previous studies (e.g., Monshouwer 2008; Reijneveld 2002; van Nieuwenhuijzen et al. 2009), namely: “Have you ever used marihuana (cannabis weed, hash, ganga)?”. The answer categories ranged from 0 = No, I have never used marihuana to 5 = Yes, every day.

#### Delinquency

Delinquency was measured with 7 items, of which most were derived from the International Self-Reported Delinquency questionnaire (ISRD; Junger-Tas et al. 1994, 2003). From this questionnaire, one item tapped vandalism (“Have you ever damaged something on purpose, such as a bus shelter, a window, a car or a seat in the bus or train?”) and four items tapped property crime related to theft. Additionally, one vandalism item from another questionnaire was also used, in addition to the item “Have you ever done something for which you were arrested by the police?” (Baerveldt et al. 2003). Thus in total, 2 vandalism items were used. The answer-categories for each of the seven items were: 0 = Never *or* Yes, but that was longer than 12 months ago; 1 = Yes, once in the past 12 months; 2 = Yes, twice in the past 12 months; 3 = Yes, three times or more during the past 12 months. A mean score was computed, with higher means reflecting higher levels of delinquency. The Cronbach alpha of 0.78, indicated adequate reliability.

#### Sensation seeking

Finally, sensation seeking was used as a control variable, since this construct has been consistently shown to be related to real-world risk-taking (Schonberg et al. 2011). It was assessed with four items of the *fun seeking* sub-scale of the Behavioral Approach System questionnaire (BAS; Carver and White 1994). This Fun seeking sub-scale is often used to measure sensation seeking tendencies (Zuckerman 2012; Franken and Muris 2006, Ko et al. 2008). An example item is “I crave excitement and new sensations”. Answers categories ranged from 1 = “Very false for me” to 4 = “Very true for me”. Cronbach’s alpha was 0.56, which is on the lower side, and this could perhaps be attributed to the small number of items on that scale.

#### Strategy of analyses

Descriptive analyses were conducted in SPSS and the main analyses were conducted in Mplus 7.11 (Muthén and Muthén 1998–2012). In Mplus, dependency within the group triads was accounted for, by using the “Type = COMPLEX” feature to adjust for standard error biases caused by the clustered data (participants clustered in groups of 3) (Korendijk et al. 2012). A Full Information Robust Maximum Likelihood Estimator (MLR) was used (Satorra and Bentler 1994) in order to adjust for any non-normality and to allow the inclusion of incomplete data. Furthermore, any item-missing data were handled using Full Information Maximum Likelihood (FIML) (Muthén and Muthén 1998–2012).

A path model was specified per risk-taking behavior in Mplus, while controlling for gender, age and sensation-seeking. Specifically, per model risky decision making (stoplight game) was simultaneously regressed on the self-reported risk-taking behaviors and the control variables age, gender and sensation seeking. All models had a perfect fit to the data (just identified).

## Results

### Study One

#### Main effects model

The mean percentage risky decision making was 33.87 (*SD* = 23.18) for the peer condition and 33.45 (*SD* = 21.60) for the alone condition, with a Cohen’s *d* of 0.02. Structural equation models accounting for dependency within the peer condition showed that peer presence did not predict risky decision making (*β* = −0.01; *p=* 0.85). No main effect of gender (*β* = −0.11; *p* = 0.07) was found. However, adolescent phase was significant (*β* = 0.22; *p* < 0.01) indicating that middle adolescents take more risks than early adolescents.

#### Peer presence by gender interaction model

**Fig. 1:**
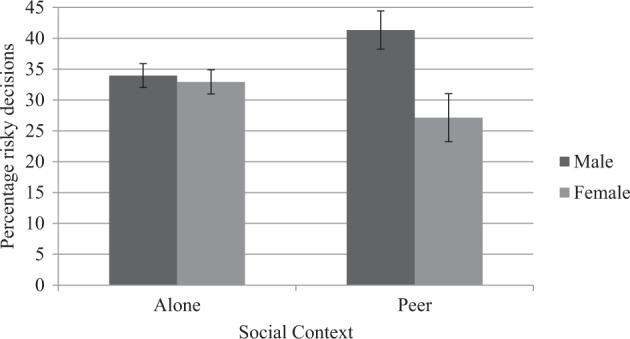
Graph of the peer presence by gender interaction. Error bars indicate standard errors of the mean

A significant interaction effect for peer presence and gender on risky decision making was found (*β* = −0.20; *p=* 0.04; Fig. 1). Follow-up post-hoc analyses showed that whereas boys and girls engage in equal levels of risk-taking (*β* = −0.02; *p=* 0.74) in the alone condition, boys significantly (Wald *χ2* (1) = 4.32; *p* = 0.04) engage in more risk-taking than girls (*β* = −0.31; *p=* 0.02) in the peer condition. Although peers did not increase risk-taking in boys (*β* = 0.12; *p=* 0.14) or girls (*β* = −0.14; *p=* 0.15), the significant interaction effect shows that peer presence has an opposite effect on male versus female risk-taking. That is, peers have an increasing effect on boys’ risk-taking, but a diminishing effect on girls’ risk-taking.

Taken together, the results of study one suggest that adolescents who perform the stoplight game together with two-same sex peers do not significantly engage in more risk-taking compared to when adolescents complete the stoplight game alone. Furthermore, gender moderated the peer presence effects on risk-taking, namely, whereas boys and girls engaged in equal levels of risks in the alone condition, boys engaged in more risk-taking than girls in the peer condition.

#### Peer presence by adolescent phase interaction model

No significant interaction effect for peer presence and adolescent phase on risky decision making was found (*β* = −0.06; *p=* 0.60). Hence, no support was found for the hypothesis that particularly early adolescents engage in heightened risk-taking in the presence of peers.

### Study Two

Bivariate correlations (Table 1) showed that risky decision making on the stoplight game was significantly correlated with all of the self-reported risk-taking behaviors, with correlations ranging from 0.12 to 0.22. The means and SD’s of risky decision making on the stoplight game, alcohol, smoking, marijuana, delinquency, sensation seeking, and traffic risk-taking were: 33.43 (21.93), 0.56 (1.11), 0.64 (1.24), 0.14 (0.60), 0.09 (0.28), 2.78 (0.53), 1.11 (0.95), respectively.

**Table 1:** Correlations of variables of interest

	1	2	3	4	5	6	7
Stoplight	–						
Alcohol	0.22^**^	–					
Smoking	0.16^**^	0.55^**^	–				
Marijuana	0.12^*^	0.42^**^	0.53^**^	–			
Delinquency	0.14^**^	0.29^**^	0.38^**^	0.59^**^	–		
SS	0.09	0.19^**^	0.23^**^	0.20^**^	0.20^**^	–	
Traffic	0.14*	0.13*	0.23**	0.18**	0.25**	0.18**	–

*SS* sensation seeking

*p < 0.05; **p < 0.01

Risky decision making on the stoplight game predicted risky traffic behavior (*β* = 0.11; *p* = 0.046), risky alcohol use (*β* = 0.15; *p* = 0.01), and delinquency (*β* = 0.12; *p* < 0.01). However, no links were found from risky decision making to smoking (*β* = 0.11; *p* = 0.06) and/or marijuana use (*β* = 0.07; *p* = 0.07).

Taken together, these results showed that risky decision making on the stoplight game was predictive of risky traffic behavior, alcohol use and delinquency in adolescents, and these linkages were found above and beyond (significant) effects of sensation seeking, age and gender (Table 2).

**Table 2:** Relations between risky decision making on the stoplight game and real-world risk behavior

	Beta	SE	*p-*value
Alcohol			
Stoplight	0.15	0.06	0.01
SS	0.13	0.05	<0.01
Age	0.32	0.05	<0.01
Gender	0.02	0.05	0.65
Delinquency			
Stoplight	0.12	0.04	<0.01
SS	0.17	0.04	<0.01
Age	0.05	0.04	0.20
Gender	−0.05	0.06	0.46
Marijuana			
Stoplight	0.07	0.04	0.07
SS	0.17	0.04	<0.01
Age	0.15	0.04	<0.01
Gender	−0.00	0.06	0.95
Smoking			
Stoplight	0.11	0.06	0.06
SS	0.18	0.05	<0.01
Age	0.22	0.05	<0.01
Gender	−0.01	0.05	0.88
Traffic			
Stoplight	0.11	0.06	0.046
SS	0.13	0.06	0.02
Age	0.12	0.05	0.03
Gender	−0.10	0.06	0.07

### Sensitivity Analyses

The interaction analyses for adolescent phase by peer presence were also conducted with adolescent phase as a continuous age variable, in order to test whether the dichotomization of age into adolescent phase (i.e., into early- versus middle-adolescence groups) influenced the results. No significant interaction effect was found for those sensitivity analyses, which is consistent with the above-described results wherein age was dichotomized. Preference was given for reporting the results of the dichotomized interaction analyses, because they test the adolescent phase moderation hypothesis more directly.

As described above, in the alone condition, the adolescents were in the same room, but behind their own computers performing the stoplight game alone and they were wearing headphones to hear the sound effects of the task (this can be referred to as a “collective” alone condition). As noted, the peer presence condition (versus this collective alone condition) did not increase risk taking. To rule out that the peer presence effect was not found because there were other adolescents in the room for the “collective” alone condition, additional data for an “individual” alone condition was collected three years later among a smaller group of adolescents. For this additional “individual alone condition”, adolescents were in a room alone while completing the stoplight game. Sensitivity analyses were conducted to double-check whether this “individual” alone condition would lead to similar results as the above-described “collective” alone condition when performance on the task is compared to the peer condition. Consistent with the results based on the collective alone condition, these sensitivity analyses showed that peer presence also did not increase risk taking in the peer condition when the individual alone condition was used. In the current article we report the results using the collective alone condition because: (1) it provided an adequate test of the current hypotheses, (2) its data was collected during the same period as the data for the peer presence condition, and (3) it had a substantially larger sample size than the individual alone condition, which made it possible to more reliably test for gender and adolescent phase moderation effects.

## Discussion

Social neurodevelopmental imbalance models posit that peer presence causes heightened adolescent risk-taking (Somerville, et al. 2011; Steinberg 2008). Whereas social neurodevelopmental imbalance models suggest that such peer presence effects particularly occur during early adolescence (e.g., Crone and Dahl 2012; Somerville et al. 2010), evolutionary theory suggests that these effects would be most pronounced in males (Wilson and Daly 1985). However, the small but growing number of experimental studies on peer presence effects in adolescent risky decision making showed mixed findings, and the vast majority of such studies did not test for the above-described gender and adolescent phase moderation effects. By taking such gender and adolescent phase moderation effects into account, the current article aimed to add to the literature about what is known about peer presence effects, and to potentially reconcile the mixed findings (study one). Furthermore, the current article assessed whether the employed laboratory risky decision-making task (i.e., the stoplight game) is meaningful for understanding real-word adolescent risk behavior (study two).

### Study One

Results of study one showed that peer presence generally did not lead to an increase in adolescent risky decision making, which contradicts the peer presence hypothesis of social neurodevelopmental imbalance models. However, there was an interaction effect between gender and peer presence on risky decision making. Follow-up post hoc analyses showed that whereas boys’ and girls’ risk-taking in the alone condition did not significantly differ, in the peer condition boys significantly took more risks than girls. Moreover, whereas same-sex peers have an increasing effect on boys’ risk-taking, same-sex peers have a diminishing effect on girls’ risk-taking. These gender moderation effects of the influence of peer presence on risk-taking are in line with evolutionary perspectives. In accordance with the current findings, one of the few experimental risk-taking studies that investigated gender differences also did not find a peer presence effect for risky decision making on the stoplight game when boys and girls were combined (Kretsch and Harden 2014). Unlike the current study, Kretsch and Harden (2014) did not investigate a moderating role of gender in peer effects, however. Nevertheless, the gender by peer presence moderation effect on experimental risk-taking is similar to the findings of another experimental study—also conducted in The Netherlands—that found that adolescent boys engaged in more risk-taking than adolescent girls in a condition wherein they completed a risky task together with peers, but not when they completed the same risky task alone (de Boer et al. 2017). Hence this finding led the authors to conclude that males appear to be more susceptible to peer influence on risk-taking compared to females (de Boer et al. 2017).

As for the theoretical framework, considering that no general peer presence effect was found, could imply that the social neurodevelopmental imbalance model might be most meaningful for adolescent boys’ heightened risk-taking in the presence of peers, but not for girls. This assertion is consistent with the *sociobiological theory of male competitiveness* (Wilson and Daly 1985) and other evolutionary perspectives on factors that influence males’ reproductive success through enhancing social reputation and dominance (Ellis et al. 2012). In line with these perspectives, the current findings perhaps suggest that since males associate their fitness with being successful in risky “competitive” situations^1^, the adolescent males (versus females) in the current study likely felt more pressured in the presence of their same-sex peers to engage in risks in order to maintain or enhance their reputation. Such peer pressure could have been transferred both verbally (directly) or non-verbally (indirectly/subtle) (e.g., Defoe et al. 2018; Wilson and Daly 1985). Equally possible is that both boys and girls encourage risk-taking, however girls are more capable of suppressing or resisting peer pressure than boys are. It should be noted however, that in the current study same-sex peers had an increasing effect on boys’ risk-taking, whereas same-sex peers had a diminishing effect on girls’ risk-taking. Thus perhaps the girls’ triad primarily consisted of pressure *discouraging* risk-taking whereas the boys’ triad consisted primarily of pressure *encouraging* risk-taking. In any case, in correlational studies, adolescent girls report more resistance to peer influence than do adolescent boys on self-report measures of peer resistance (Steinberg and Monahan 2007), and peers have been shown to have more negative influences on boys’ risk-taking compared to girls’ risk-taking (Mears et al. 1998; Piquero et al. 2005). Thus these correlational results are consistent with the current results that show a gender moderation effect of peer influence on risk-taking.

Taken together, consistent with *evolutionary perspectives on why males take more risks* and the aforementioned past correlational studies, the present results could suggest that whether peer presence sensitizes adolescents to rewards leading to risk-taking and/or whether this sensitization to respond to the rewarding aspect of risk-taking behaviors further undermines self-regulation capacities (e.g., resistance to peer influence) (Albert et al. 2013), might further be modulated by gender. However, this effect existed using just one type of risky decision-making task (driving task), and although such effects were also found on the BART (de Boer et al. 2017), it is worthwhile for future studies to explore whether these moderation effects are also found for other types of risky decision-making tasks. Finally, peer presence effects might also be modulated by the abovementioned social mechanisms (e.g., peer pressure or peer norms). Thus future studies could further explore whether social learning perspectives could be relevant for understanding peer presence effects on risk-taking.

Next, inspired by social neurodevelopmental imbalance models it was expected that particularly early adolescents would be most susceptible to peer presence effects on risk-taking. However, no age moderation effects of peer presence existed in the current study. Perhaps, the age discrepancy between early adolescents and mid-adolescents was not large enough to capture such effects. For example, a comparison between early versus mid-late adolescents would have been a more pronounced difference in adolescent phase and could have provided more power for identifying adolescent phase moderation effects in peer presence effects on risk-taking (see e.g., Steinberg and Monahan 2007). Future studies could explore this possibility with a sample with wider age ranges.

### Study Two

Study two demonstrated that the above-described results of study one are meaningful for understanding real-word adolescent risk-taking behaviors. Namely, overall performance on the stoplight game predicted risky traffic behavior, alcohol use and delinquency in adolescents, and these linkages were found above and beyond effects of sensation-seeking, age, and gender. The stoplight game is a simulated risky driving task, thus it is to be expected that performance on this task predicted self-reported real-world risky traffic behavior—and this speaks to its criterion validity. With regard to alcohol use, the current findings are more or less consistent with Kim-Spoon et al. (2016). However, that study included older (i.e., late) adolescents, and the present results further suggest that the significant link found in that study from performance on the stoplight game to a composite score of smoking, alcohol and marijuana, might be primarily driven by alcohol use. All things considered, the current findings suggest that perhaps the decision-making processes that are at play during completion of the stoplight game, are the same underlying processes that contribute to real-world risky traffic behavior, alcohol use and delinquency in adolescents.

### Strengths, Limitations, Implications and Future Directions

Several limitations should be considered when interpreting the current findings. Namely, although the current study only focused on two basic potential moderators of the peer presence effect on risk-taking, there are multitudes of other relevant factors that could moderate the peer presence effect. For example, a recent review on laboratory risk-taking suggests that the peer presence might particularly lead to heightened risk-taking, when peers are deviant (Defoe et al. 2019). The deviant peer presence effect has even been captured in the few experimental studies that investigated it (see: Paternoster et al. 2013; Mercer et al. 2018). Hence, it is recommended that future studies investigate the peer presence effect, but to also consider using diverse alone and peer paradigms when doing so. For example, peer paradigms in which risk-taking is encouraged versus discouraged (i.e., negative versus positive peer pressure) or paradigms wherein close friends versus mere peers are used, could increase the understanding of *when* peer presence increases or decreases risk-taking. Paradigms wherein peer pressure is present, and wherein close friends are used could also be ecologically stronger (but see de Boer et al. 2017), and thus might mirror real-world risk-taking scenario’s better than the current employed paradigms.

Relatedly, studies in more natural settings could be additionally informative compared to the laboratory settings (i.e., classroom settings) used in the current study. For example, it cannot be concluded for certain whether the findings in the current laboratory settings could be transferred to settings in the real-world (e.g., at a party) where risk-taking behaviors typically occur. Nevertheless, the current study also examined self-reported real-world risk behaviors, and it has further showed that performance on the stoplight game in a laboratory setting is related to real-world risk behaviors above and beyond significant effects of sensation-seeking. Thus, the current study provides some new insights into the predictive power of a laboratory task on multiple self-reported real-world risk-taking behaviors in adolescents, and therefore adds to the literature in a significant way as criterion validity is not typically assessed in experimental studies. However, when interpreting the results readers should keep in mind that the effect sizes were modest.

## Conclusion

Heightened risk behavior during adolescence typically occur when adolescents are with their peers (Steinberg 2008). Recent experimental studies that have investigated this hypothesized peer presence effect on heightened adolescent risk-taking have yielded inconsistent results, however. The current study investigated whether such inconsistent results might stem from neglected gender and/or adolescent phase moderation of peer presence effects on adolescent risk-taking. The current results showed no general peer presence effect on heightened adolescent risk-taking, but instead peer presence effects only existed for boys. These results contradict *social neurodevelopmental imbalance models* that do not posit gender differences, however they do support the evolutionary theories on gender differences in peer influence on risk-taking. Namely, they suggest that heightened adolescent risk-taking in the presence of peers might be gender specific, for both early- and mid-adolescents. In the real-world, adolescent boys evidently engage in more (antisocial) risk-taking behaviors than adolescent girls (e.g., Moffitt and Caspi 2001), however the current experimental study raises an interesting observation that this gender difference might particularly arise when adolescent boys are in the company of same-gender peers. Taken together, the present findings demonstrate that individual differences (i.e., gender) could determine how the social environment (i.e., peer presence) could affect adolescent risk-taking. Furthermore, the current study demonstrated that the stoplight game has adequate criterion validity, as it predicted heightened adolescent real-world risk-taking behaviors such as risky traffic behavior, alcohol use and delinquency above and beyond predicting sensation-seeking. The finding that performance on a laboratory risky decision-making task can perhaps help identify adolescents that are vulnerable to heightened risky traffic behavior, alcohol use and delinquency, is an important finding for science as well as prevention and intervention efforts.

## Footnotes

1 This was the case in the current study, considering that before participants began the stoplight game, they were told that “a prize would be given to the person in their school who finishes the game the fastest” (please see the Methods section).
